# Retraction Note: AKT3-mediated IWS1 phosphorylation promotes the proliferation of EGFR-mutant lung adenocarcinomas through cell cycle-regulated *U2AF2* RNA splicing

**DOI:** 10.1038/s41467-022-31445-7

**Published:** 2022-06-28

**Authors:** Georgios I. Laliotis, Evangelia Chavdoula, Maria D. Paraskevopoulou, Abdul Kaba, Alessandro La Ferlita, Satishkumar Singh, Vollter Anastas, Keith A. Nair, Arturo Orlacchio, Vasiliki Taraslia, Ioannis Vlachos, Marina Capece, Artemis Hatzigeorgiou, Dario Palmieri, Christos Tsatsanis, Salvatore Alaimo, Lalit Sehgal, David P. Carbone, Vincenzo Coppola, Philip N. Tsichlis

**Affiliations:** 1 Department of Cancer Biology and Genetics, The Ohio State University, Columbus, OH USA; 2 The Ohio State University Comprehensive Cancer Center-Arthur G. James Cancer Hospital and Richard J. Solove Research Institute, Columbus, OH USA; 3 School of Medicine, University of Crete, Heraklion, Crete Greece; 4 Molecular Oncology Research Institute, Tufts Medical Center, Boston, MA USA; 5 Department of Clinical and Experimental Medicine, Bioinformatics Unit, University of Catania, Catania, Italy; 6 Department of Medicine, Division of Hematology, The Ohio State University, Columbus, OH USA; 7 Tufts Graduate School of Biomedical Sciences, Program in Genetics, Boston, MA USA; 8 DIANA-Lab, Hellenic Pasteur Institute, Athens, Greece; 9 Institute of Molecular Biology and Biotechnology, Foundation for Research and Technology Hellas, Heraklion, Crete Greece; 10 Department of Internal Medicine, Division of Medical Oncology, The Ohio State University Medical Center, Columbus, OH USA; 11 Present Address: Department of Oncology, Sidney Kimmel Comprehensive Cancer Center, Johns Hopkins School of Medicine, Baltimore, MD USA; 12 Present Address: Center of Basic Research, Biomedical Research Foundation of the Academy of Athens, Athens, Greece; 13 Present Address: Department of Pathology, Beth Israel-Deaconess Medical Center, Harvard Medical School, Boston, MA USA

**Keywords:** Non-small-cell lung cancer, RNA splicing

Retraction to: *Nature Communications* 10.1038/s41467-021-24795-1, published online 30 July 2021.

The authors are retracting this Article as irregularities were found in the data that indicate the splicing of the U2AF2 exon 2 does not occur as reported in the Article. The irregularities call into question the conclusions and undermine their confidence in the integrity of the study. The authors therefore wish to retract the article.

All authors agree with the retraction.

